# A guide to do randomized controlled trials in the field of otolaryngology

**DOI:** 10.1186/s43163-021-00142-5

**Published:** 2021-07-21

**Authors:** Sameh M. Zamzam, Mosaad Abdel-Aziz, Ahmed Atef, Usama Abdel-Naseer, Mostafa Hamoda, Mohamed Salah, Hazem Dewidar, Louay Elsharkawy, Mena E. Abdelmalek

**Affiliations:** ENT Department, Kasr Alainy Hospital, Garden city, Cairo, Egypt

**Keywords:** Study design, Randomized clinical trials, Otolaryngology, Research

## Abstract

**Background:**

Randomized controlled trials (RCTs) are prospective comparative studies in which study groups are allocated randomly to intervention or serve as controls. RCT is the mainstay to achieve evidence in the literature in clinical research. A RCT is the main research design to study the effect of an intervention and the only way to confirm the value of a new treatment.

**Main body:**

RCT also gives the way to generate meta-analyses and systematic reviews giving a stronger evidence for clinical practice. Evidence-based medicine (EBM) is crucial for safe, effective, and standardized patient care. Although there is an agreement on the importance of performing RCT, it can be challenging to do it efficiently including different aspects like study design, funding, randomization, blinding, follow-up, data analysis, statistics, generalization of results, and reporting of quality of the studies.

**Conclusion:**

In this article, we gave a comprehensive review for RCT in otolaryngology discussing their importance, advantages, and drawbacks, types, steps, challenges, reporting their quality and their prevalence in the literature.

## Background

RCT is a study in which subjects are randomly assigned into groups including a control group, to receive or not receive intervention under study. The study results are determined by comparing outcomes of interest between different groups and their statistical analysis to determine whether the difference is clinically significant or not. The terms “randomized control trial” and “randomized trial” are usually used interchangeably. However, “randomized control trial” refers to the comparison between intervention and control groups while “randomized trial” refers to the comparison between multiple intervention groups [1].

Sir Bradford Hill (1897–1991) was the first to publish RCT in medicine. He is a British epidemiologist and statistician and is considered to be the father of modern RCT. There is a progressive increase in the number of published articles in otolaryngology. This opened the way for more evidence-based treatment recommendations and well-settled guidelines. However, this provided new challenges for otolaryngologists including continuous review for updates in the published research, ability to assess its quality, provide continuously updated treatment guidelines, and individualizing patient care according to the most recent available evidence in the literature [2, 3].

## Main text

### Importance of RCTs

Evidence from RCTs and subsequently systematic reviews lies at the top of the pyramid of evidence and is considered the most important source for evidence-based clinical decisions. On contrary, in observational studies, there are often differences in characteristics between study groups, this will result in bias because the outcome may differ due to such differences not due to the intervention itself. The only way to overcome this bias is to randomly allocate individuals to the desired intervention or control [4].

This article gives an overview of the different types and steps you can follow to do randomized controlled trials. It also focuses on their advantages and drawbacks and how to avoid these mistakes during the study. Furthermore, it gives a quick review about reporting the quality of RCTs and the prevalence of RCTs in otolaryngology in recent decades.

### Types of randomized clinical trials

- Randomized controlled clinical study: Evaluates therapeutic agents like drugs, e.g., evaluation of levocetirizine in treating allergic rhinitis.
- Preventive study: Study primary prevention methods, e.g., coronavirus disease-19 (COVID-19) vaccination.
- Risk factor study: Intervention to stop a risk factor that shares in the development of a disease, e.g., treatment of single laryngeal papilloma to protect against laryngeal cancer.
- Cessation study: Evaluate the cessation of a habit causing a disease, e.g., alcohol and cancers of the larynx and pharynx.
- Etiologic agents’ study: To confirm or reject the possible role of the etiologic factor for a disease.
- Evaluation of health system: To evaluate the effectiveness of the health system in the prevention and treatment of certain diseases to reduce the burden on national costs, e.g., the effectiveness of specific hospital infection control measures to reduce the incidence of intensive care unit (ICU) morbidity [5].

### Types of controls in randomized controlled clinical trials

- Placebo control: Study participants in the control group receive treatment that contains no active drug.
- No treatment control: Study participants in the control group receive no treatment, so they are not blind to treatment allocation.
- Active (positive) control: Study participants in the control group receive an active treatment, e.g., managing otitis media with effusion with ventilation tube insertion versus medical treatment.
- Dose response control: The study investigates the effect of different doses of the same treatment so different groups are given different doses, e.g., investigating optimal steroids dose in Bell’s palsy.
- External control: The control group used is outside the current study, being treated at an earlier time (historical control) or in another setting.
- Multiple control groups: Using more than one type of the previously mentioned controls [6].

### Advantages and drawbacks of RCTs

#### Advantages

- Effective in eliminating selection bias and controls confounding bias without adjustment due to similar characteristics of study groups.
- Control of exposure to intervention; including time, amount, frequency, and duration.
- High statistical power can be achieved.
- Allows comparison of multiple outcomes.
- Optimal for publication.

#### Drawbacks

- Complex, expensive, time-consuming, sometimes ethically questionable.
- Artificial environment, if strict eligibility criteria are applied, the generalizability of results will be questionable.
- Select subjects who will comply to the treatment regimen and strict follow-up, this may compromise the generalizability of results.
- Cannot be used for rare outcomes or outcomes needing very long follow-up [7].

### Steps of conducting a RCT design

#### Formulating study question

A RCT is generally used to test an intervention, which needs to be verified, exploring its safety and effectiveness. The proposed intervention should be safe and ethically approved. Observational studies can provide an idea about the effect of a specific intervention, but they are generally liable to bias. RCTs are used to study treatment methods and diagnostic tools but they are not appropriate or practical for studying etiology, natural history of disease, rare outcomes, or outcomes that take a very long time to occur [8].

Study hypothesis can be classified into:

- Superiority study: An intervention is supposed to be better than another one and the difference is statistically significant.
- Non-inferiority study: An intervention is supposed to be not worse than a reference intervention in a statistically significant way.
- Equivalence study: Two interventions are supposed to be equal in statistical significance [9].

RCTs can be classified according to outcome into:

- Explanatory study: Explores if the study intervention works and its mechanism.
- Pragmatic study: In addition to studying if the interventions work, it assesses all the results of the intervention and its use under real situations of the clinical practice.
- Efficacy study: Tests the effect of intervention on the people receiving it.
- Effectiveness study: Tests the effect of intervention in the people offered the intervention [5].

#### Selection of participants

##### Inclusion and exclusion criteria

Inclusion criteria aim to identify the patients in whom the intervention is appropriate and likely to lead to the tested outcome. Included subjects should be representative of the population of interest and inclusion criteria should not be too restricted so that the generalizability of the results is affected. Exclusion criteria should include patients in which risk of treatment toxicity is unacceptable, likely ineffective, having comorbidities interfering with the intervention, or unlikely to comply with the intervention and follow-up. In general, there should be a balance between choosing the appropriate participants without being too restricted at the expense of the generalizability of results. Both inclusion and exclusion criteria are very significant in minimizing sampling or referral bias [10].

#### Sample size

According to the number of participants, RCTs are classified into:

- Fixed trials: Fixed number of subjects is calculated using statistical methods before the start of the intervention.
- Mega trials: Include thousands of patients from multiple centers. This increases statistical power and generalizes the results.
- Sequential trials: The number of subjects is not specified. The researchers continue recruiting participants until a clear outcome.
- Individual patient trials include one patient. The results cannot be generalized.

Adequate sample size and power are important to be able to generalize the study conclusions to the targeted population with confidence. This number is often determined by previous trials, observations, experiments, or consensus opinion. Specific statistical methods are used to obtain a valid number. In general, when the group characteristics are less variable, a smaller group size will be needed to prove that the difference is mostly not due to chance and is most probably due to the intervention. A very small sample size increases the chance that the effect of the intervention won't be significant, even if it has a real effect (type 2 error). A type I error refers to the rejection of the null hypothesis while it is true, a type II error refers to the failure of rejection of the null hypothesis while it is false [11].

#### Trial phases

Clinical trials assessing pharmaceutical agents should run a course of studies to determine if they are safe and effective before being permitted for clinical use. There are four steps of clinical trials to be completed.

- Phase I: Studying drug pharmacology in human, like studying the absorption, excretion, and toxicity. They are done in a small number of volunteers nearly 20–80.
- phase II: Exploring therapeutic efficacy, estimating dose, and confirming the safety in a slightly larger number of participants nearly 100–300.
- Phase III: Confirming the therapeutic effect of the drug, using adequate sample size for proper confirmation before clinical use of the drug.
- phase IV: Done after drug approval by health authorities like FDA or European medicines agency as post-marketing studies to give more data about uses, side effects, and optimizing dose [12].

#### Baseline measurements

- Describe subjects’ demographics: age, sex, etc. They may especially important to emphasize that the randomization process worked.
- Participants’ contact information that helps in avoiding loss of follow-up during the study including phone number, home address, or contact information of a friend or family member to contact if the participant is not reachable.
- Identify prognostic factors of the outcome that can be identified, e.g., if a side effect of the drug is thought to be more common in females, so collecting data on gender is crucial [6].

#### Randomization

Important to eliminate confounding and selection bias and balance prognostic variables in all groups.

##### Criteria of randomization

- Unpredictability: Giving the same chance for every study subject to be allocated to any of the interventions or control groups.
- Equality: Ensuring that study groups have the same number and baseline characteristics.
- Simplicity: Choosing an easy way to be done by the researcher [13].

##### Methods of randomization

- Simple randomization: Randomization is like flipping a coin. The most common methods are random number table and randomization computer programs to generate random numbers. It is simple and easy but a number of subjects in each study group may be different especially in small studies [13].
- Stratified randomization: Control and balance the influence of covariates. Subjects are firstly divided into blocks of covariates then simple randomization is done within each block to allocate subjects to the study groups. It may be particularly useful with a small number of subjects. No need for stratified randomization in large clinical trials because participants’ characteristics are adequately balanced in study groups [14].
- Block randomization: Especially useful with small sample size to provide an equal size of study groups all time. The block size should be a multiple of the number of study groups (e.g., if there are 2 study groups, block size can be 4, 6, or 8). The main disadvantage is that the participants randomized in the last of the block can predict their study group, so they will not be blinded to the selection and its application is very difficult [15].
- Unequal randomization: Like allocating fewer number of participants to the more expensive intervention and more number to the cheaper one. The results are usually of low statistical power [15].

#### Blinding

It is important to eliminate measurement bias.

##### Types of blinding

- Open: Study participants, investigators, and statistician know each participant is allocated to which intervention or control (not blinded).
- Single-blind: Study participants or investigators are blinded to the intervention, but not both of them.
- Double-blind: Study participants and investigators are blinded to the intervention.
- Triple-blind: Participants, investigators, and statisticians are blinded [16].

#### Intervention

According to participants’ exposure to the intervention, RCTs can be classified into:

- Parallel: The most commonly used type, treatment, and control interventions are given to different individuals e.g. studying endoscopic myringoplasty and comparing it to microscopic myringoplasty as control. They generally require larger sample size to produce statistically significant results.
- Crossover: Every participant receives both treatments, but their order is randomized. Therefore, each participant is considered his own control, and subsequently less sample size is needed.
- Factorial: It allows investigating the joint effect of two or more factors together, e.g., in Reinke’s edema, one can study the effect of medical treatment alone versus surgery versus both together versus control. It also facilitates the study of interactions.
- Cluster: Randomization is done to a whole group of subjects, e.g., allocating the intervention to a whole hospital or department and control to another hospital or department. It can be used to assess interventions that cannot be applied to separate subjects like comparing the effect of specific infection control measures on the incidence of certain hospital infections [17].

#### Follow-up of subjects

Compliance to protocol and avoidance of follow-up loss are important aspects for RCT. Loss of follow-up, non-compliance, and contamination (Patients cross from one study group to the other) are important potential problems in RCTs. They will lead to reduced statistical power due to decreased subjects. If they occurred in a non-random manner, i.e., in a group more than the other, which is often the case, they will lead to bias [18].

#### Measurement of outcome

- Primary and secondary outcomes are assessed.
- Positive results/negative results could be obtained.
- Adverse events are reported
- Intention-to-treat analysis: a method to analyze results in RCT, for each group separately regardless of the received treatment in each group’s participants [3].

#### Statistical analysis

Statistical analysis of RCTs is usually straightforward as all factors other than the intervention itself are balanced, allowing accurate comparison of the study outcomes between different groups. The *t* test is usually used for continuous outcomes, while the chi-square test is used for categorical outcomes. Non-parametric tests may be used if the sample size is small or when the outcomes are not normally distributed. In survival studies, the Kaplan-Meier curve or advanced COX regression model is used to be able to estimate the outcome over time. If the population parameters such as mean and standard deviation (SD) of the present study are claimed to be equal to previous data analysis, this is known as the null hypothesis (H_0_); on the other hand, the alternative hypothesis (H_1_) is the actual data analysis of the present study [19].

### Non-randomized controlled trials

Unlike RCTs, study participants are not assigned to interventions or control in a random way but according to the decision of the investigator, so characteristics of participants are not similar across study groups. To rightly assess the effect of the intervention, differences in participants’ characteristics between different groups should be controlled in data analysis. If not done, it usually overestimates the advantages of one intervention over another.

It may be used instead of RCT in the following situations:

- When it may decrease the effectiveness of the intervention.
- When it would be unethical or illegal.
- When it is impractical (e.g., costly) [20].

### Reporting quality of RCTs

RCTs are considered the best way for “rational therapeutics” in medical practice. There may be a big difference in quality between different RCTs. To standardize the method of reporting quality of RCTs and include all important aspects in the evaluation process, a team of scientists created the CONSORT (Consolidated, Standards Of Reporting Trials) statement. It is a checklist and flow diagram that include all important aspects for the quality of the RCT including for example the study design, randomization, blinding, and results. It is now the most agreed method for reporting the quality of RCTs [21].

### Prevalence of RCTs in the field of otolaryngology in the last 2 decades

Yao et al. in 2007 reviewed all RCTs of treatment efficacy in 6 years from 2000 to 2005 in 4 major otolaryngology journals in relation to the volume of published research. Banglawala et al. in 2015 reviewed all RCTs of treatment efficacy in three years from 2011 to 2013 in the same journals, compared with the previous study, there is an approximate doubling in volume of yearly published articles, but there is a decrease in the percentage of published RCTs. Banglawala et al. concluded that RCTs represent a small percentage out of the whole published research in otolaryngology; however, the quality and reporting of RCTs are improving (Table 1) [22, 23].

**Table 1:** Prevalence of RCTs in the field of otolaryngology in the last 2 decades

Journal	Yao et al. 2007 [22]		Banglawala et al. 2015 [23]	
	Years 2000–2005		Years 2011-2013	
	Number of RCTs/total number of articles	Number of RCTs	Number of RCTs/total number of articles	Number of RCTs
*The Annals of Otology, Rhinology and Laryngology*	202/5467 (3.7%)	15 (7%)	189/5729 (3.3%)	24 (13%)
*Archives of Otolaryngology— Head and Neck Surgery (JAMA)*		50 (25%)		26 (14%)
*The Laryngoscope*		86 (43%)		74 (39%)
*Otolaryngology—Head and Neck Surgery*		51 (25%)		65 (34%)

## Conclusion

In this article, we gave a comprehensive review for RCT in otolaryngology discussing their importance, advantages, drawbacks, types, steps, challenges, reporting their quality, and their prevalence in the literature.

This current study gives a step-by-step guide to do a RCT in the field of otolaryngology when compared to previous similar studies.

RCT has chief characters: unpredictable distribution and equality of participants in both groups, high statistical power which makes it optimal for publication. The main cornerstones of RCT are to determine the control group, sample size, method of randomization, type of blinding, and exposure.

RCT should be limited to settings and subjects with characteristics similar to the study settings and subjects.

Future studies are needed to assess the prevalence of RCT in different topics in the field of otolaryngology in different local, regional, and international journals.

## Data Availability

Data are available from the corresponding author on reasonable request

## Abbreviations

RCT: Randomized clinical trial/randomized controlled trial
EBM: Evidence-based medicine
